# Pyr1-Mediated Pharmacological Inhibition of LIM Kinase Restores Synaptic Plasticity and Normal Behavior in a Mouse Model of Schizophrenia

**DOI:** 10.3389/fphar.2021.627995

**Published:** 2021-03-12

**Authors:** Sylvie Gory-Fauré, Rebecca Powell, Julie Jonckheere, Fabien Lanté, Eric Denarier, Leticia Peris, Chi Hung Nguyen, Alain Buisson, Laurence Lafanechère, Annie Andrieux

**Affiliations:** ^1^ Department of Molecular and Cellular Neurosciences, Grenoble Institute Neuroscience, Inserm U1216, Grenoble, France; ^2^ Université Grenoble Alpes, Grenoble, France; ^3^ Health Department, Interdisciplinary Research Institute of Grenoble, CEA, Grenoble, France; ^4^ Chimie et Modélisation pour la Biologie du Cancer, Institut Curie, PSL Research University, CNRS UMR9187, Inserm U1196, Orsay, France; ^5^ Microenvironment, Cell Plasticity and Signaling Department, Institute for Advanced Biosciences, CNRS UMR5309, Inserm U1209, Grenoble, France

**Keywords:** MAP6, LIM kinase, actin, microtubule, therapeutics, cytoskeleton, cognitive abilities

## Abstract

The search for effective treatments for neuropsychiatric disorders is ongoing, with progress being made as brain structure and neuronal function become clearer. The central roles played by microtubules (MT) and actin in synaptic transmission and plasticity suggest that the cytoskeleton and its modulators could be relevant targets for the development of new molecules to treat psychiatric diseases. In this context, LIM Kinase - which regulates both the actin and MT cytoskeleton especially in dendritic spines, the post-synaptic compartment of the synapse - might be a good target. In this study, we analyzed the consequences of blocking LIMK1 pharmacologically using Pyr1. We investigated synaptic plasticity defects and behavioral disorders in MAP6 KO mice, an animal model useful for the study of psychiatric disorders, particularly schizophrenia. Our results show that Pyr1 can modulate MT dynamics in neurons. In MAP6 KO mice, chronic LIMK inhibition by long-term treatment with Pyr1 can restore normal dendritic spine density and also improves long-term potentiation, both of which are altered in these mice. Pyr1 treatment improved synaptic plasticity, and also reduced social withdrawal and depressive/anxiety-like behavior in MAP6 KO mice. Overall, the results of this study validate the hypothesis that modulation of LIMK activity could represent a new therapeutic strategy for neuropsychiatric diseases.

## Highlights

• LIMK inhibition stabilizes the neuronal cytoskeleton
• LIMK inhibition normalizes dendritic spine density *in vitro* and *in vivo*
• Pyr1 treatment improves long-term hippocampal synaptic transmission and plasticity
• Pyr1 reduces social withdrawal and depressive/anxiety-like phenotypes in MAP6 KO mice

## Introduction

Our knowledge of the brain defects associated with neuropsychiatric disorders has only started to increase since the start of the second millennium, thanks to the development of new techniques and advances in imagery. We know that the neuronal cytoskeleton plays a fundamental role in the plasticity of synaptic connections, both at the pre-synaptic axonal boutons and at the post-synaptic dendritic spines, which are the membrane protrusions emanating from neuronal dendrites (Dent, 2020). Much work initially focused on the function and regulation of actin filaments in dendritic spines, but more recently microtubules (MTs) originating from the dendritic shaft were shown to enter into spines to regulate their activity and morphology (Jaworski et al., 2009; Hotulainen and Hoogenraad, 2010; Kapitein et al., 2010). In accordance with these key roles, synaptic cytoskeletal alterations can lead to cognitive defects with both abnormal neuronal connectivity and defective synaptic plasticity. These cytoskeleton-related neuronal defects are landmarks of psychiatric disorders, in particular schizophrenia (Benitez-King et al., 2004; Fromer et al., 2014; Hall et al., 2015; Marchisella et al., 2016). For example, proteins encoded by genes mutated in schizophrenia such as dysbindin, DISC1 (disrupted one in schizophrenia) or CRMPs (Collapsin Response Mediator Proteins) were shown to localize to cytoskeleton-rich regions in neurons and to regulate actin and MT dynamics (Morris et al., 2003; Talbot et al., 2006; Ujike et al., 2006; Lin et al., 2011; Wang and Brandon, 2011; Bader et al., 2012; Khazaei et al., 2014; Quach et al., 2015; Niwa et al., 2017). In patients with schizophrenia, LIM Kinase (LIMK) was also found to be deregulated (Datta et al., 2015; Zhao et al., 2015), and a direct molecular link was established between LIMK and neuregulin 1, which is encoded by a schizophrenia-susceptibility gene (Yin et al., 2013). In accordance with their links to schizophrenia, LIMKs are present in dendritic spines where they modulate dendritic spine morphology and synaptic functions, roles that have considerable influence during synaptic plasticity events (Meng et al., 2002; George et al., 2015; Wang and Townes-Anderson, 2016). The deregulation of LIMKs expression in schizophrenia (Datta et al., 2015; Zhao et al., 2015) makes them attractive targets for therapeutic intervention through the use of small pharmacological compounds (Shaw and Bamburg, 2017). Although few pharmacological regulators of LIMKs have been identified (Prunier et al., 2016; Manetti, 2018), Pyr1 was found to be highly selective and, in cycling cells, capable of stabilizing MTs and modulating actin dynamics (Prudent et al., 2012).

The LIM Kinase family is composed of two highly related members, LIMK1 and LIMK2. LIMKs, in particular LIMK1, were first identified as the factor causing the cognitive defects observed in Williams-Beuren syndrome (Hoogenraad et al., 2004; Gray et al., 2006). At the cellular level, LIMKs regulate actin filament dynamics through phosphorylation and inactivation of cofilin (Bamburg et al., 1999) as well as MT dynamics through an as yet incompletely identified mechanism (Gorovoy et al., 2005; Prudent et al., 2012; Bhardwaj et al., 2014).

For this study, we hypothesized that by regulating the neuronal cytoskeleton’s dynamics, Pyr1 might work as a pro-cognitive drug affecting synaptic plasticity and thus modulating behavior. To test this possibility, we analyzed the effect of Pyr1 on biological and behavioral defects in MAP6 KO mice (also known as STOP KO mice), a pertinent mouse model used to study psychiatric disorders. Indeed, MAP6 KO mice constitutively exhibit behavioral and biological features relevant to some aspects of psychiatric disorders, including schizophrenia and major depressive disorder. Among others phenotypes, MAP6KO mice display severe social withdrawal (Andrieux et al., 2002; Bégou et al., 2008; Volle et al., 2013), anhedonia and reduced motivation (Fournet et al., 2012; Fournet et al., 2012), and anxiety/depression-like behaviors (Jonckheere et al., 2018) as well as memory defects, cognitive deficits, and impaired sensory-gating abilities (Fradley et al., 2005; Powell et al., 2007; Volle et al., 2013). At the biological level, MAP6 KO mice have diminished neurogenesis (Fournet et al., 2010), impaired synaptic plasticity in the hippocampus associated with totally defective long-term potentiation (Andrieux et al., 2002; Delotterie et al., 2010), and a reduced density of dendritic spines (Peris et al., 2018). Importantly, the behavioral and biological defects in MAP6 KO mice are partially alleviated by long-term treatment with the gold-standard pharmacological treatments for psychiatric disorders, i.e., antipsychotics and antidepressants (Andrieux et al., 2002; Bégou et al., 2008; Delotterie et al., 2010; Fournet et al., 2012). Similar improvements were obtained with the animal equivalent of electro-convulsive therapy (Jonckheere et al., 2018), which has a high rate of success with treatment-resistant depressive disorders in humans. In addition, and interestingly for this study, as an indication that cytoskeletal modulation might help alleviate neuronal defects related to psychiatric disorders, the MT modulators epothilone D (a Taxol-like component) and Davunetide (a peptide derived from the Activity Dependent Neuroprotective Protein, ADNP), were also shown to alleviate behavioral and biological defects in MAP6 KO mice, particularly those linked to synaptic impairments (Andrieux et al., 2006; Merenlender-Wagner et al., 2010; Fournet et al., 2012; Merenlender-Wagner et al., 2014).

The results presented here demonstrate that chronic LIMK inhibition by treatment with Pyr1 restores synaptic plasticity and alleviates some behavioral defects in MAP6 KO mice. Pyr1 thus behaves like a neuroactive molecule with possible pro-cognitive capacity.

## Materials and Methods

### Cell Culture, Viral Infection, and Immunofluorescence

Neurons from WT or MAP6 KO embryos were obtained as previously described (Peris et al., 2018). Briefly, hippocampi from WT and MAP6 KO littermate embryos (E17.5 or 18.5) were dissected and digested in 0.25% trypsin for 15 min. After manual dissociation, neurons were plated on poly-L-lysine-coated cover slips (for viral infection and immunofluorescence) or on petri dishes (for western blot analysis). Newly-isolated neurons were incubated for 2 h in DMEM-10% horse serum, medium was then changed to MACS medium (Miltenyl Biotec) supplemented with B27 (Invitrogen).

#### Visualization of Dendritic Spines

1/100 of a hippocampal neuron suspension was infected by incubating for 20 min with GFP lentivirus at a multiplicity of infection of 40 cells were then plated on poly-l-lysine-coated coverslips. Neurons were cultured for 17 days *in vitro* (17 DIV) before treatment with dimethyl sulfoxide (DMSO, untreated) or 185 nM Pyr1, for 30 min, fixation in PFS (4% sucrose, 4% Paraformaldehyde in PBS) and successively labeled with rabbit polyclonal anti-GFP antibody (1/2,000, Invitrogen) and anti-rabbit antibody coupled to AF 488 (1/1,000, Jackson Immuno-Research Laboratories).

#### Analysis of Microtubule Stability

MAP6 KO neurons at 10 DIV were treated with DMSO (vehicle control) or 10 μM Pyr1 for 30 min and then exposed to cold (45 min, 4°C). WT neurons were exposed or not to cold (45 min, 4°C). Cells were permeabilized (30 mM Pipes, 1 mM EGTA, 1 mM MgCl_2_, 10% glycerol, 1% Triton X 100, pH 6.75) for 1 min and fixed in cold methanol (6 min, − 20 C). For immunofluorescence, anti-tubulin mAb was used as primary antibody (clone α3A1, 1/2000, 45 min) and binding was revealed with anti-mouse coupled to AF 488 (1/1000, Jackson Immuno-Research Laboratories). To quantify MT stability, a 50 µm diameter circular ROI (corresponding to 7,850 µm^2^) was drawn on the nucleus of isolated neurons (Hoechst image) and the surface occupied by MT network was measured in the ROI after segmentation (threshold for positive signal was set at 3 standard deviation above the background mean intensity).

### Western Blot Analysis of Cofilin Phosphorylation

Neurons at 10 DIV (plated on poly-l-lysine-coated 35 mm petri dishes) were treated with DMSO (untreated condition) or 20 μM Pyr1 for 30 min. Plates were rinsed before harvesting cells by scraping in lysis buffer (50 mM Tris, 150 mM NaCl, 1% NP40, 0.5% DOC, pH 8) in the presence of protease and phosphatase inhibitors (Complete Cocktail tablet and PhosSTOP tablets, Roche). Cell lysates were separated on SDS-PAGE and transferred to PVDF-LV membrane (Biorad) for immunoblotting with rabbit polyclonal anti-phospho-cofilin (P-Cof, Ser3, Cell Signaling) or anti-cofilin (Cof, Cell Signaling), together with anti-tubulin (clone α3A1). Anti-Rabbit coupled to AF 488 and anti-mouse coupled to AF647 were used as secondary antibodies (1/2,000, Jackson Immuno-Research Laboratories). Blots were analyzed on a ChemiDoc imaging system (Biorad). P-Cof and Cof signals were normalized relative to the signal for tubulin on the same blot.

### Animals and Pharmacological Treatment

#### Ethical

The study protocol was approved by the local animal welfare committee (Comité Local GIN, C2EA-04 - APAFIS number 8303-2016060110523424) and complied with EU guidelines (directive 2010/63/EU). Every precaution was taken to minimize the number of animals used and stress to animals during experiments.

#### Animals

Homogeneous inbred C57BL6/129SvPas-F1 MAP6 KO and MAP6 KO/Thy-eYFP mice were obtained as previously described (Deloulme et al., 2015). All experiments were performed using 2–5 month-old males, and mice were carefully matched for age across treatment groups. Researchers performing procedures were blinded to the animal’s treatment group.

#### Pyr1 Treatment

Pyr1, synthesized as described (Prudent et al., 2012), was diluted in NaCl-PEG solution (0.432% NaCl, 32% PEG400) from a 50 mg/ml stock solution in dimethyl sulfoxide (DMSO). Pyr1 was injected intraperitoneally, twice a week at a dose of 100 mg/kg/week for at least 6 weeks.

### Quantification of Dendritic Spine Density

Dendritic spine density was assessed in the motor cortex (layer V, bregma 1.18 mm) of MAP6 KO Thy-eYFP-H mice, or in primary cultures of hippocampal neurons infected with lvGFP (see cell culture section), as previously described (Peris et al., 2018). Briefly, confocal images of dendritic segments (apical secondary or tertiary neurites in the cortex and primary or secondary neurites in cell culture) were obtained using a laser-scanning microscope (Zeiss, LSM 710) fitted with a X63 oil-immersion objective. Optical sections, with pixel dimensions of 0.083 μm × 0.083 μm, were collected at 200 mm intervals. Dendritic spine density was analyzed in 3D in the z-stack using NeuronStudio software (Rodriguez et al., 2008). Dendric spines were classified using the default settings in NeuronStudio. Between one and three neurites were measured for each neuron with a total length measured exceeding 50 µm. Images acquisition and analysis were done blind to genotype and to experimental conditions.

### Measuring Hippocampal Neurotransmission and Synaptic Plasticity

#### Ex Vivo Slice Preparation

Brain slices were prepared from 3 month-old littermates, either control animals or treated with Pyr1. The brain was removed quickly, and 350 μm sagittal slices containing both cortex and hippocampus were prepared in ice-cold sucrose solution (2.5 mM KCl, 1.25 mM NaH_2_PO_4_, 10 mM MgSO_4_, 0.5 mM CaCl_2_, 26 mM NaHCO_3_, 234 mM Sucrose, and 11 mM Glucose, saturated with 95% O2 and 5% CO_2_) with a Leica VT1200 blade microtome (Leica Microsystemes, Nanterre, France). The hippocampus was dissected from the slice and transferred to oxygenated artificial cerebrospinal fluid (ACSF; 119 mM NaCl, 2.5 mM KCl, 1.25 mM NaH_2_PO_4_, 1.3 mM MgSO_4_, 2.5 mM CaCl_2_, 26 mM NaHCO_3_, 11 mM Glucose) at 37 ± 1°C for 30 min, and then kept at room temperature for at least 1 h before recording neurotransmission.

#### Electrophysiological Recordings

Each slice was individually transferred to a submersion-type recording chamber and continuously superfused (2 ml/min) with oxygenated ACSF. Extracellular recordings were taken in the apical dendritic layers of the hippocampal CA1 area, using glass micropipettes filled with ACSF. Field excitatory post-synaptic potentials (fEPSPs) were evoked by the electrical stimulation of Schaeffer collaterals afferent to CA1. The magnitude of the fEPSPs was determined by measuring their slope. Signals were acquired using a double EPC 10 Amplifier (HEKA Elektronik Dr. Schulze GmbH, Germany), recorded with Patchmaster software (HEKA Elektronik Dr. Schulze GmbH, Germany) and analyzed using Fitmaster software (HEKA Elektronik Dr. Schulze GmbH, Germany). Input/output (I/O) curves: the slope of fEPSPs was plotted as a function of stimulation intensity (0–100 µA). Paired-Pulse Facilitation (PPF) of synaptic transmission was induced by paired stimulation with different interstimulus intervals; from 25 to 300 ms. PPF was quantified by normalizing the magnitude of the second response relative to the magnitude of the first response. For long-term potentiation (LTP), test stimuli were delivered once every 15 s, adjusting the stimulus intensity to produce 40–50% of the maximal response. A stable baseline was recorded for at least 15 min. LTP was induced by high-frequency stimulation (100 Hz stimulation for 1 s, repeated twice with 20 s between each train). The average fEPSP slope value was expressed as a percentage of the baseline response.

### Behavioral Tests

#### Social Interaction Test

Home-cage social interaction was assessed based on the duration of sniffing investigation displayed by a resident mouse in response to presentation of an anesthetized intruder mouse (to avoid the aggressive component (Yamada et al., 2000). In this test, resident untreated and Pyr1-treated MAP6 KO mice were individually housed for 5 days before testing to encourage home-cage territory behavior. Intruder mice (same age and unfamiliar to resident mice) were anesthetized by i.p. injection with ketamine (100 μg/g) 10 min before starting the experiment. The intruder was placed at the center of the resident’s home cage (L39 cm × W9 cm × H13 cm), and the resident’s exploration activity was immediately video-recorded for 5 min. Exploration activity was quantified as the time spent sniffing the intruder animal.

#### Novelty Suppressed Feeding Test (NSF)

The NSF test is an anxiety-based conflict test where the motivation to eat competes with fear of a brightly-lit area (Santarelli et al., 2003; David et al., 2009). The NSF test was performed for 15 min. Briefly, animals were prepared for the test by fasting, induced by removing access to food in their home cage for 20 h. For the test, a single food pellet was placed on a brightly-lit white paper platform positioned in the center of a plastic box (L37 cm × W57 cm × H20 cm), the floor of which was covered with approximately 2 cm of bedding. The animal was placed in a corner of the box and their latency to eat was recorded.

### Statistical Analysis

Statistical analyses were performed using Prism 8 software (GraphPad). Bilateral Mann-Whitney *U* test or Student's *t*-test were used to determine the statistical significance of differences, as indicated in figure legends.

## Results

In this study, MAP6 KO mice were used as a model of schizophrenia and the impact of long-term treatment with Pyr1, a LIMK inhibitor and a modulator of MT/actin dynamic (Prudent et al., 2012), on MAP6 KO mice defects, was analyzed in terms of behavioral, anatomic and physiological parameters.

### LIM Kinase Inhibition Stabilizes the Neuronal Cytoskeleton

**FIGURE 1:**
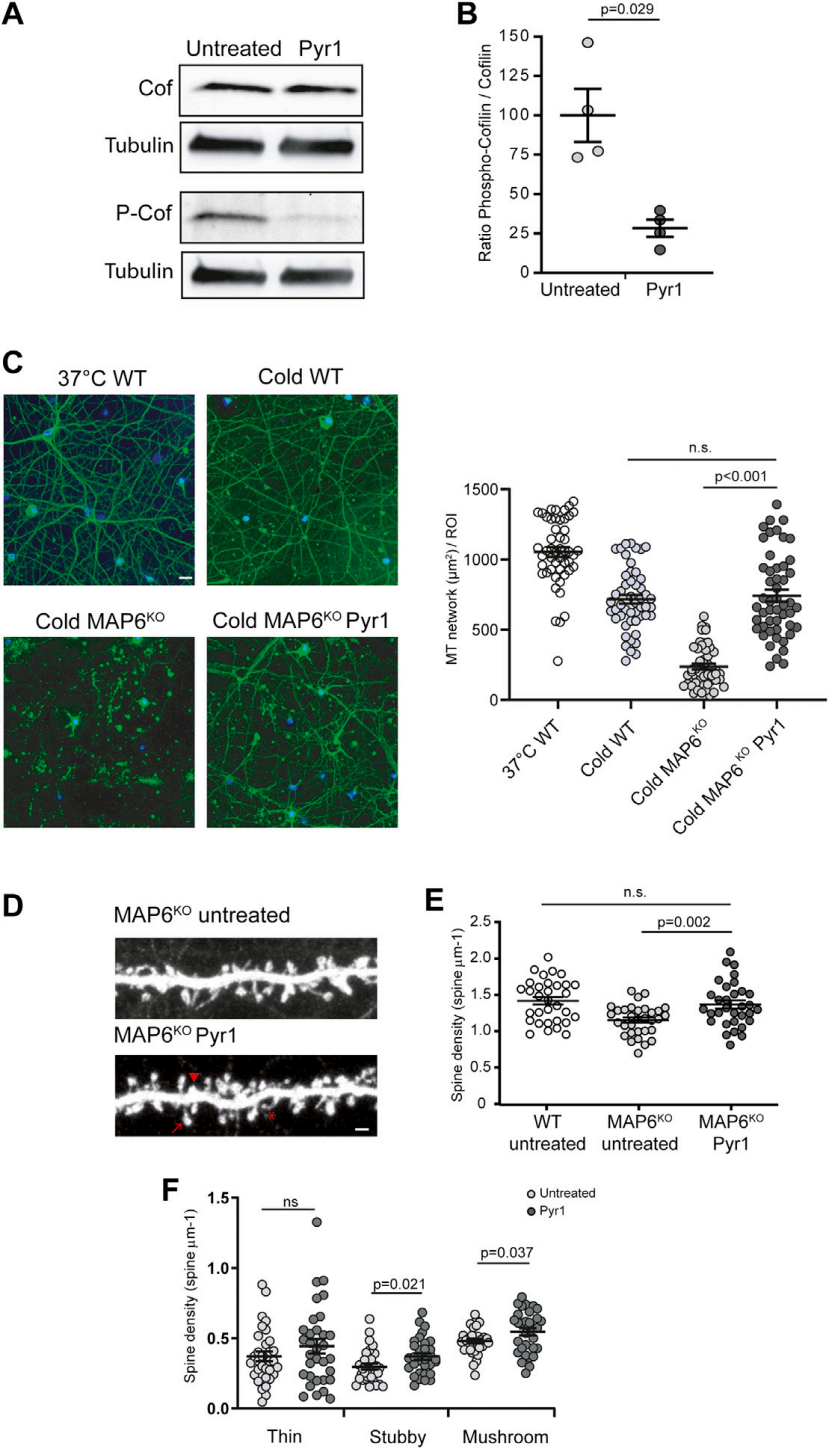
Pyr1 modulates cytoskeleton dynamics and neuronal dendritic spine density **(A,B)** Effect of Pyr1 on cofilin phosphorylation. Hippocampal neurons, untreated or treated with 20 μM Pyr1 for 30 min were lysed, and proteins analyzed by western blot. The relative amounts of cofilin and phosphocofilin were assessed. **(A)** Representative western blot with samples from untreated and Pyr1-treated neurons. **(B)** Quantification of phosphocofilin/cofilin ratio, values are expressed as a proportion of the mean for untreated samples (100 ± 16.8 for untreated, 28.4 ± 5.4 for Pyr1 treated neurons). Mann-Whitney test, *p* value is indicated, *n* = 4 independent experiments **(C)** Effect of Pyr1 on MT stability. Wild type neurons were either non-cold exposed (37°C WT) or cold-exposed (cold WT). Untreated MAP6 KO neurons and Pyr1-treated MAP6 KO neurons (30 min, 10 µM Pyr1, 37°C), were exposed to cold (0°C for 45 min). Following free tubulin extraction, cells were permeabilized and MT were labeled using anti-tubulin antibody (green); nuclei were stained with Hoechst (blue). Scale bar = 20 µm. Quantification of MT surface was performed in each condition. MT surface per ROI were 1053 ± 34 for 37°C WT; 717 ± 31 for cold WT, 237 ± 20 for cold MAP6 KO and 742 ± 42 for cold MAP6 KO Pyr1-treated. Student’s t-test, *p* values are indicated, ns: not significant. *n* = 50 neurons in each condition **(D–F)** Effect of Pyr1 on dendritic spine density on MAP6 KO neurons **(D)** Confocal image showing representative examples of dendritic segments of MAP6 KO hippocampal neurons infected with lvGFP at 17 DIV to visualize dendritic spines. Representative spines sub-types as classified by Neuron Studio software, are illustrated: star = thin spine, arrow head = stubby spine and arrow = mushroom spine. Untreated MAP6 KO or Pyr1-treated neurons are shown. Scale bar = 2 μm Pyr1 treatment consisted in the application of 185 nM Pyr1 for 90 min to the culture. Dendritic spine density was quantified. For total spines **(E)**, densities were 1.41 ± 0.05 for WT neurons, 1.15 ± 0.04 and 1.34 ± 0.05 spines/µm for untreated KO and treated KO neurons, respectively. For classified spines **(F)** densities were: 0.37 ± 0.04 and 0.44 ± 0.05 thin spines/µm; 0.30 ± 0.02 and 0.37 ± 0.02 stubby spines/µm; 0.48 ± 0.02 and 0.55 ± 0.03 mushroom-like spines/µm for untreated and Pyr1-treated neurons, respectively. Student's t-test, *p* values are indicated, ns: not significant. *n* = 30 neurons from three independent neuronal cultures for MAP6 KO neurons, *n* = 32 neurons for WT neurons, from four independent cultures. One to three neurites were measured for each neuron, total length ≥50 µm. Values correspond to mean ± SEM.

LIMK-mediated phosphorylation of cofilin results in its inactivation. The ratio between inactive and active cofilin (*p*-Cof/Cof) will subsequently affect the actin dynamics in the cell. We therefore monitored the levels of phospho-cofilin (*p*-Cof) and cofilin (Cof) in hippocampal neurons grown for 17 days *in vitro* and exposed to Pyr1 for 30 min, and compared them to levels measured in control cultures. As shown in Figure 1A, Pyr1 treatment induced a decrease in p-Cof expression. Results from four independent experiments using MAP6 KO neurons indicated a 72% decrease in the p-Cof/Cof ratio (Figure 1B). Similar results were obtained using wild type or MAP6 heterozygous neurons (Supplementary Figure S1). These results indicate that Pyr1 effectively reduces LIMK activity in neurons, and the reduced level of cofilin phosphorylation should modulate the dynamics of neuronal actin. Notably, no difference in the amounts of Cof, *p*-Cof, LIMK and phospho-LIMK was found between MAP6 KO and WT mice (Supplementary Figure S1).

In cycling cells Pyr1 has been shown to regulate MTs (Prudent et al., 2012), so we next examined the impact of Pyr1 on MT dynamics/stability in neurons. For these tests, we exploited the fact that, as previously described (Andrieux et al., 2002), in contrast to wild type neurons (Figure 1C), MAP6-KO neurons are sensitive to cold exposure, which results in almost complete MT depolymerization (Figure 1C). When MAP6 KO neurons were exposed to Pyr1 prior to cold exposure, the MT network was preserved (Figure 1C). After cold exposure, the surface occupied by MTs was strongly diminished in untreated MAP6 KO neurons as compared to WT neurons (Figure 1C). For Pyr1-treated MAP6 KO the MT network is preserved with a surface occupied by MT non-significantly different from WT values (Figure 1C). In control experiments we showed that cold exposure does not affect the actin organization in neurons (Supplementary Figure S2). These results indicate that Pyr1-mediated LIMK inhibition leads to stabilization of neuronal MTs.

### LIM Kinase Inhibition Normalizes Dendritic Spine Density *In Vitro* and *In Vivo*

MT and actin dynamics are important for the plasticity of dendritic spines, and LIMK1 was shown to control actin dynamics in spines (George et al., 2015). We thus wondered whether inhibition of LIMKs by Pyr1 could alleviate the reduced dendritic spine density observed in MAP6 KO neurons (Peris et al., 2018). MAP6 KO neurons treated with Pyr1 increased their total spine density by 29% compared to untreated neurons (Figures 1D,E), 1.34 ± 0.05 spines/µm for Pyr1-treated MAP6 KO, as compared to 1.15 ± 0.04 spines/µm for untreated MAP6 KO neurons), reaching values non-significantly different to those recorded for WT neurons (1.41 ± 0.05 spines/μm). Dendritic spines can be classed in three morphological types: thin, stubby, and mushroom-like spines. In these *in vitro* experiments, Pyr1 treatment specifically enhanced the density of stubby and mushroom-like spines (Figure 1F), which correspond to the mature and functional spines. These results indicate that LIMK inhibition can restore normal dendritic spine density in MAP6 KO neurons through modulation of spine plasticity.

These findings prompted us to assay Pyr1 activity on dendritic spines in the whole animal. To do so, MAP6 KO mice were treated with Pyr1 long-term (6 weeks), and compared to untreated animals. An average of 95 nM Pyr1 was detected in brains from treated mice, as assayed by LC-MS/MS, whereas no detectable Pyr1 molecule was detected in brains from untreated mice (Supplementary Figure S3).

**FIGURE 2:**
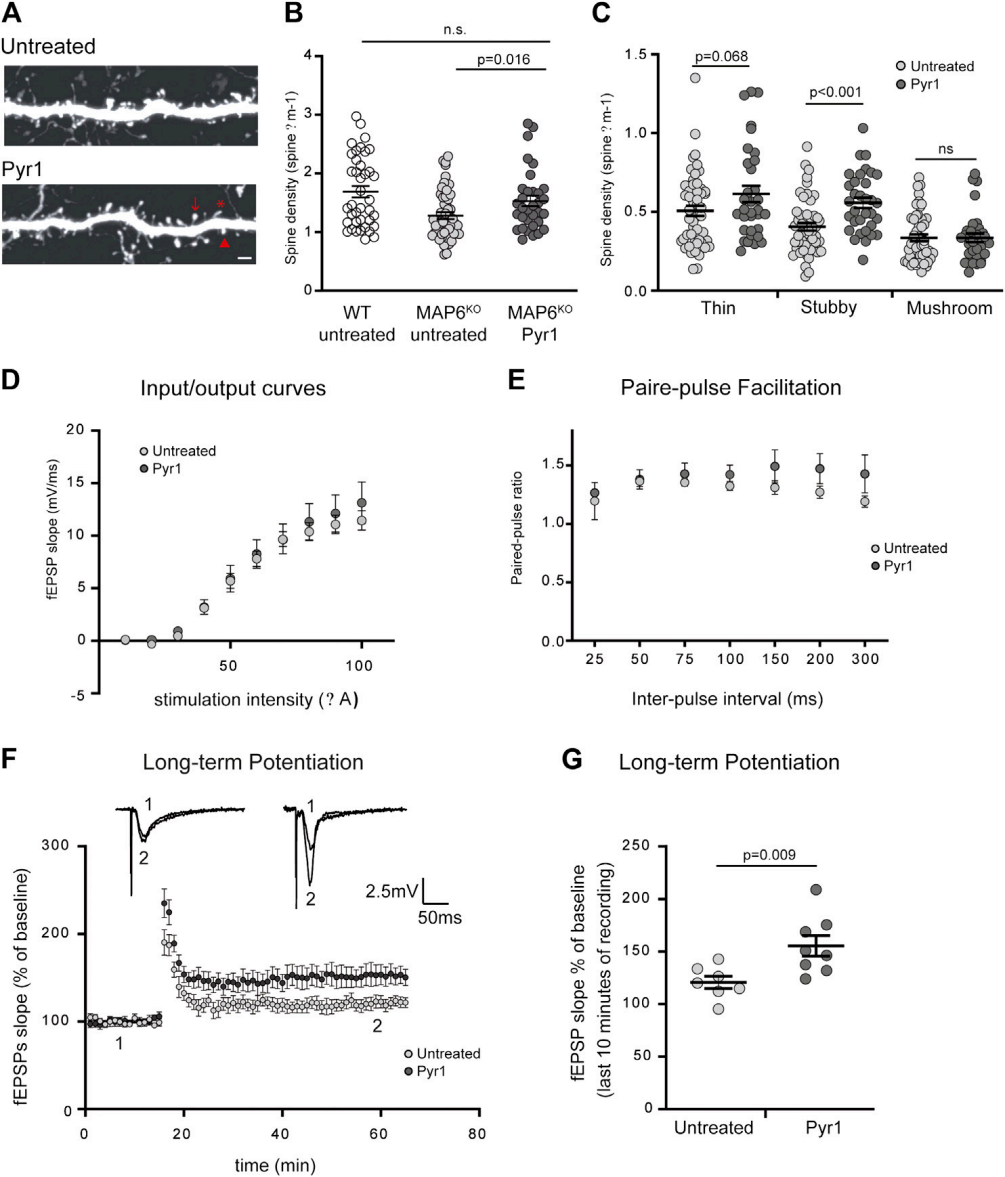
Pyr1 treatment improves defective dendritic spine density and synaptic plasticity in MAP6 KO mice. **(A–C)** Effect of Pyr1 on dendritic spine density *in vivo*. Representative confocal images of dendritic segment in MAP6 KO layer V, corresponding to cortical neurons, from untreated or Pyr1-treated (100 mg/kg/week, 6 weeks) mouse brains. Representative spines sub-types as classified by Neuron Studio software, are illustrated: star = thin spine, arrow head = stubby spine and arrow = mushroom spine **(A)**. Scale bar = 2 µm. Quantification of total dendritic spine density. For total spines **(B)** values were: 1.67 ± 0.10 for WT neurons, 1.25 ± 0.06 and 1.51 ± 0.09 spines/µm for untreated MAP6 KO and Pyr1-treated MAP6 KO neurons, respectively. For classified spines **(C)** densities were: 0.51 ± 0.03 and 0.61 ± 0.05 thin spines/µm; 0.40 ± 0.02 and 0.55 ± 0.03 stubby spines/µm; 0.33 ± 0.02 and 0.34 ± 0.03 mushroom-like spines/µm, for untreated and Pyr1-treated neurons, respectively. Values correspond to mean ± SEM. Student’s t-test *p* values are indicated. ns: not significant. *n* = 39 neurons from four WT mice, *n* = 52 neurons from six untreated mice, and *n* = 33 neurons from four Pyr1-treated. One to two neurites were measured for each neuron, total length ≥50 µm **(D–G)** Effect of Pyr1 on hippocampal synaptic transmission and plasticity in MAP6 KO mice **(D)** Basal synaptic transmission analyzed based on input-output curves generated by plotting Field EPSP slope against stimulation intensity. Recordings were performed in brain slices from untreated and Pyr1-treated mice. *n* = 5 slices from four untreated and four Pyr1-treated mice. **(E)** Short-term plasticity was monitored by measuring the Paired-Pulse Facilitation (PPF). Seven distinct inter-pulse intervals ranging from 25 to 300 ms in slices were used to measure paired-pulse ratios in slices from untreated and Pyr1-treated mice. *n* = 5 slices each from four untreated and four Pyr1-treated mice **(F–G)** Long-Term Potentiation (LTP) was assayed in the CA1 region **(F)** fEPSP slopes expressed as a percentage of baseline were plotted against time for untreated and Pyr1-treated brain slices. Representative fEPSP traces were extracted at times 1 and 2, as indicated **(G)** fEPSP slopes measured over the last 10 min of recordings, expressed as a percentage of the baseline fEPSP slope (120.8% ± 5.9 and 155.5% ± 9.8 for untreated and Pyr1-treated mice, respectively). Values correspond to mean ± SEM. Mann-Whitney test, *p* value is indicated. *n* = 7 slices from three untreated mice and *n* = 8 slices from five Pyr1-treated mice.

To visualize dendritic spines in MAP6-deficient neurons *in vivo*, MAP6 KO mice were cross-bred with Thy1-eYFP-H transgenic mice (Feng et al., 2000). In the transgenic MAP6 KO offspring, as compared to hippocampal neurons that express high level of yellow fluorescent protein, the layer V cortical neurons express moderate level allowing accurate quantification of spine density. In a previous work, MAP6 KO mice were shown to display a reduced spine density in layer V cortical neurons as compared to WT neurons (Peris et al., 2018). We thus analyzed dendritic spine density and morphology in brain cortical sections from MAP6 KO mice treated with Pyr1, or untreated, by confocal microscopy (Figure 2A). Quantification revealed an overall increase of 32% in dendritic spine density in the treated group (Figures 2B), 1.25 ± 0.06 spines/µm for untreated MAP6 KO neurons as compared to 1.51 ± 0.09 spines/µm for Pyr1-treated MAP6 KO neurons), reaching values not significantly different to those recorded for WT neurons (1.67 ± 0.10 spines/μm). Analysis of the distribution of these dendritic spines between the three morphological types indicated that density was significantly improved for mature stubby spines (Figure 2C).

These results indicate that injection of Pyr1 into the whole animal can induce *in vivo* recovery of dendritic spine density in MAP6 KO neurons. This result suggests functional recovery, and incited us to examine the effects of Pyr1 on the defective synaptic plasticity observed and characteristic behavioral phenotypes of MAP6 KO mice.

### Pyr1 Treatment Improves Long-Term Hippocampal Synaptic Transmission and Plasticity

The Pyr1-induced increase in dendritic spine density could have an incidence on synaptic plasticity, especially on LTP, a parameter that has been consistently shown to be drastically altered in MAP6 KO mice (Andrieux et al., 2002; Andrieux et al., 2006; Delotterie et al., 2010). We assessed the consequences of long-term Pyr1 treatment on overall glutamatergic synaptic transmission using extracellular field recordings in the CA1 region of the hippocampus. No detectable differences in input/output curves (Figure 2D) or in PPF ratio (Figure 2E) were found between untreated or Pyr1-treated brain slices. These results indicate that chronic Pyr1 treatment does not alter normal synaptic transmission and short-term synaptic plasticity. We next examined LTP, and as previously reported (Andrieux et al., 2002; Andrieux et al., 2006; Delotterie et al., 2010), MAP6 KO mice displayed LTP deficits (Supplementary Figure S4). A significant improvement in LTP was observed in slices from MAP6 KO Pyr1-treated mice compared to slices from untreated mice (Figure 2F). Quantification of the average fEPSP slopes over the last 10 min of recordings indicated that they were significantly increased in slices from Pyr1-treated mice (Figure 2G), 120.8% ± 5.9 and 155.5% ± 9.8 for untreated and Pyr1-treated mice, respectively), reaching values close to those recorded in WT mice (Supplementary Figure S4), 158.2% ± 10.8, for WT mice). These results suggest that chronic treatment with Pyr1 improves long-term synaptic plasticity in MAP6 KO mice.

Alleviation of MAP6 KO synaptic plasticity deficits by long-term Pyr1 treatment might lead to improvements in some of the behavioral phenotypes exhibited by MAP6 KO mice.

### Pyr1 Treatment Reduces Social Withdrawal and the Depressive/Anxiety-Like Status of MAP6 KO Mice

To determine whether the functional improvements induced by chronic treatment with Pyr1, observed at the level of neurons and of synaptic plasticity, results in behavioral ameliorations in MAP6 KO mice, we performed two independent behavioral tests.

**FIGURE 3:**
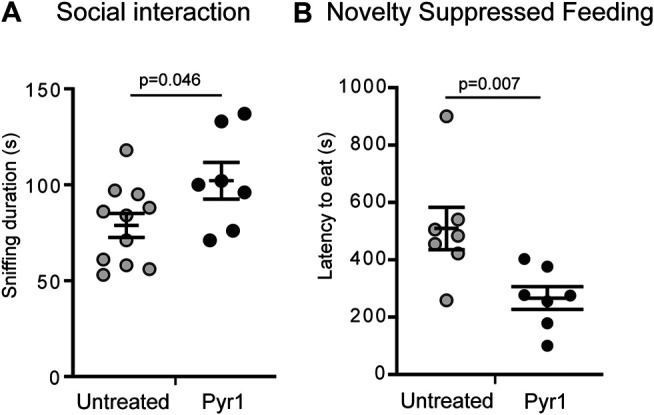
Pyr1 treatment alleviates behavioral defects of MAP6 KO mice **(A)** Effect of Pyr1 on social behavior. Social interaction was assayed by measuring the time a MAP6 KO resident male spent sniffing an intruder (78.8 ± 6.2 and 102.0 ± 9.6 for untreated and Pyr1-treated mice, respectively). Sniffing behavior was analyzed over a 5 min period. Values correspond to mean ± SEM. Mann-Whitney test, *p* = 0.046. *n* = 11 for untreated mice and *n* = 7 for Pyr1-treated mice **(B)** Effect of Pyr1 on depressive/anxiety status. Novelty Suppressed Feeding (NSF) test was used to measure the latency to eat (509.3 ± 73.6 and 266.4 ± 39.7 for untreated and Pyr1-treated mice, respectively). Values correspond to mean ± SEM. Mann-Whitney test, *p* value is indicated. *n* = 7 for untreated and Pyr1-treated mice.

Initially, as MAP6 KO mice exhibit severe social withdrawal (Bégou et al., 2008; Delotterie et al., 2010) which has been consistently shown to respond positively to pharmacological treatments, we analyzed the effect of Pyr1-treatment on this parameter. Social interaction was assessed based on the sniffing time displayed by a resident male, housed alone for 5 days, in response to the presentation of an anesthetized intruder mouse. The intruder was anesthetized to avoid aggressive responses and allow measurement of social behavior alone. As previously reported (Bégou et al., 2008; Delotterie et al., 2010), MAP6 KO mice exhibited a social interaction deficit, as measured based on sniffing time of an intruder mouse – 73 ± 6 s for MAP6 KO – compared to 105 ± 8 s for WT (Supplementary Figure S4). The social withdrawal observed in MAP6 KO mice was improved by the 6 weeks treatment with Pyr1 (Figure 3A). Following this treatment, the sniffing time duration for Pyr1-treated MAP6 KO mice was significantly longer than that recorded for untreated mice (Figures 3A), 102 ± 10 s for treated mice vs 79 ± 6 s for untreated mice). Indeed, values were similar to those recorded for WT mice (105 ± 8 s, Supplementary Figure S4).

We subsequently used the Novelty Suppressed Feeding (NSF) test to analyze the severe depressive/anxiety status of MAP6 KO mice that was recently shown to positively respond to non-pharmacological Electro-Convulso-Stimulation (ECS) (Jonckheere et al., 2018). The NSF test reveals the degree of competition between the mouse's motivation to eat and its aversion to bright light. MAP6 KO mice display a significantly higher latency to eat than WT mice (Jonckheere et al., 2018). After 6 weeks’ chronic treatment with Pyr1, the latency to eat was almost halved, lasting 266 ± 39 s, compared to the untreated mice, for which a latency of 509 ± 73 s was recorded (Figure 3B).

These behavioral tests demonstrated a beneficial effect of chronic Pyr1 treatment on the behavioral phenotypes exhibited by MAP6 KO mice.

## Discussion

The aim of this study was to analyze the effects of Pyr1, a selective LIMK inhibitor, on neuron plasticity and behavior in MAP6 KO mice, an animal model with constitutive hallmarks of psychiatric-like disorders. Using MAP6 KO primary neuron cultures, we demonstrated that Pyr1 could affect LIMK activity in these highly differentiated cells, and thus resulted in inhibition of cofilin phosphorylation. LIMK inhibition was previously shown to lead to stabilization of the microtubule network in epithelial cells (Prudent et al., 2012). Distinguishing a stable MT network from free tubulin dimers, using tubulin immuno-staining, is generally delicate (Ramirez-Rios et al., 2020), and even more so in neuronal extensions where MTs are tightly packed and difficult to distinguish. Fortunately, primary cultures of neurons from MAP6 KO mice, with their cold-sensitive MT network allowed us to demonstrate that treatment of the neurons with Pyr1 protected MT from depolymerization, unambiguously demonstrating Pyr1’s ability to stabilize neuronal MTs. The capacity of Pyr1 to modulate the dynamics of MTs thus seems to be a general feature in cells.

LIMK activity is known to be related to dendritic spine integrity. Indeed, decreased LIMK1 activity in LIMK KO neurons leads to abnormal dendritic spine morphology (Meng et al., 2002), and hippocampal neurons treated with LIMK1shRNA display decreased dendritic spine density (George et al., 2015). Our results, using either *in vitro* approaches or *in vivo* whole-animal experiments, confirmed that inhibition of LIMK in a disease-like context, somewhat unexpectedly, effectively altered dendritic spine density. We showed that Pyr1 treatment reestablished a normal density of mushroom and stubby spines in cultured neurons whereas only stubby spines are normalized *in vivo*. Both stubby and mushroom spines are considered as active spines (Runge et al., 2020). *In vivo*, the preferential increase in stubby spines, that we observed, might reflect a difficulty in the common categorization in two distinct sub-types due to the limited spatial resolution during image acquisition. Accordingly, some stubby spines could have very short necks connecting the head to the dendrite which are not been detected in our conditions (Tonnesen et al., 2014), leading to an over representation of the stubby subtype at the expense of the mushroom one. We hypothesize that, in our experimental conditions, Pyr1 did not completely block LIMK but rather modified the equilibrium between its active and inactive forms. Consequently, LIMK activity appears to have been maintained within a defined and optimal range, whereas total inactivation or full activation would be deleterious. This observation stresses the importance and the relevance of pharmacological tools for the modulation of LIMK activity, as they can be used to fine-tune LIMK activity by dose-adaptation.

The positive action of LIMK inhibitor on dendritic spine integrity in MAP6 KO neurons might involve actin. Indeed, it has been shown that, in the absence of MAP6 protein, actin dynamics are altered in synapses during plasticity events. During such events, and particularly LTP, the amount of cofilin in spines increases rapidly (Bosch et al., 2014); the severing of actin filaments by cofilin creates new barbed ends for actin nucleation, and thus promotes actin polymerization (Van Troys et al., 2008). LIMK inhibition would increase the amount of active cofilin, and could thus restore the functional equilibrium of actin dynamics, explaining why normal LTP is restored in MAP6 KO mice following Pyr1 treatment, especially in the early stages. These results are in agreement with the recent demonstration that pharmacological control of actin cytoskeleton dynamics, by modulating the balance between globular and filamentous actin, can either promote or prevent synaptic plasticity (Medina et al., 2020).

The positive action of LIMK inhibitor on dendritic spine integrity in MAP6 KO neurons might also be a result of Pyr1’s capacity to stabilize MTs. Indeed, MTs have been detected that extend from the dendritic shaft into the dendritic spine (Hu et al., 2008; Jaworski et al., 2009). Adapting microtubule stability might affect microtubule residence time in spines. Accordingly, our previous results demonstrate that the microtubule stabilizing molecule epothilone D (Epo D) can restore impaired synaptic plasticity in MAP6 KO mice (Andrieux et al., 2006). In addition, Epo D, as well as the 8-amino-acid peptide NAP (Davunetide) - which has been shown to act through interaction with MTs (Divinski et al., 2004) - improve cognitive behavior in MAP6-deficient mice (Andrieux et al., 2006; Merenlender-Wagner et al., 2010; Fournet et al., 2012; Merenlender-Wagner et al., 2014).

We recently reported that certain 12 bis-aryl urea derivative inhibitors of LIMK (Yin et al., 2013) had a stabilizing effect on cellular microtubules (Ramirez-Rios et al., 2020). Interestingly, treatment with the 12 bis-aryl urea derivative SR7826, which is also a LIMK inhibitor, rescued β-amyloid-induced hippocampal spine loss and morphological aberrations (Henderson et al., 2019). Future research should compare the effects of this compound to those of Pyr1.

In addition to the modulation of actin and MTs dynamics, the positive effects of Pyr1 on LTP could also involve other LIMK substrates known to be crucial for synaptic plasticity, such as CREB and CPEB (Prunier et al., 2017). At the molecular level, a better understanding of the regulation of LIMK activity, the respective roles of the two isoforms, and how they regulate microtubules will shed light on the mechanisms involved in the effects described here.

In the context of psychiatric disorders, both in preclinical animal models and in humans, the beneficial effects of antidepressants, antipsychotics, and Electro-Convulsive Treatment (ECT) have been shown, at least partly, to involve an enhancement of adult hippocampal neurogenesis, with an increase in the proliferation of neuronal progenitors (Jonckheere et al., 2018; O'Leary and Cryan, 2014). Our investigations into a possible effect of Pyr1 on the defective adult neurogenesis observed in MAP6 KO mice (Fournet et al., 2010) revealed no effect on the proliferation rate of hippocampal progenitors (Supplementary Figure S5). This result suggests that MAP6 KO behavioral defects can be alleviated through action on cellular pathways shared by other effective treatments, but also on distinct pathways. Overall, the results presented here indicate that the use of molecules modulating cytoskeleton dynamics (LIMK inhibitor, epothilone or NAP), in combination with a classical antipsychotic or antidepressant treatment could be a relevant pharmacological treatment for schizophrenia or major depression.

Altogether, our results strongly support a treatment approach involving precise modulation of cytoskeleton dynamics to induce improvements in synaptic plasticity and alleviate the cognitive defects which characterize psychiatric disorders and the early stages of most neurodegenerative diseases.

## Data Availability

The raw data supporting the conclusions of this article will be made available by the authors, without undue reservation.
