# Supplementary material for: Pyr1-Mediated Pharmacological Inhibition of LIM Kinase Restores Synaptic Plasticity and Normal Behavior in a Mouse Model of Schizophrenia
Source: Front Pharmacol. 2021 Mar 12;12:627995. doi: 10.3389/fphar.2021.627995 (PMC8006432; doi:10.3389/fphar.2021.627995)
Supplement: Supplementary file 1 [file datasheet1.pdf]

Supplementary Figure S1

*Effect of Pyr1 on cofilin phosphorylation

Hippocampal neurons from Wild Type (WT) or MAP6 heterozygous (He) mice were incubate with or without 20µM of Pyr1 during 30 min, lysed and proteins analyzed by western blot (as in Fig1 A).

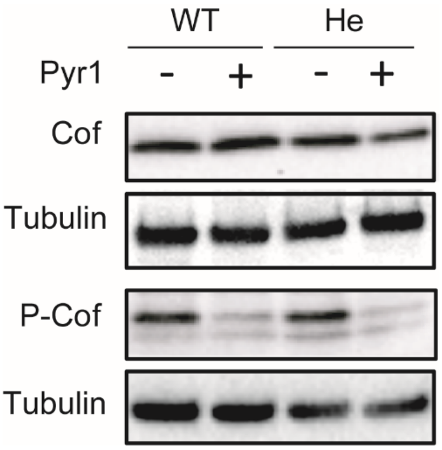

Pyr1 treatment induces a decrease of P-Cof in WT and MAP6 He neurons.

*Level of Cofilin and LIMK in mice cortices

Cortices of WT and MAP6 KO mice were lysed in HEPES-NP40 buffer (50mM Hepes pH7, 150mM NaCl, 5 mM MgCl2, 5mM CaCl2) supplemented with proteases and phosphatases inhibitors. Lysates were analyzed with similar protocol as in Fig 1B. Antibodies used were rabbit polyclonal anti-cofilin (1/2000, Cell Signaling), anti-phospho Cofilin (1/2000, P-Cof, Ser3, Cell Signaling), anti LIMKinase (1/1000, Cell Signaling), anti-phospho LIMK (1/1000, thr508, Abcam). Quantification of protein were normalized on tubulin and expressed as a % of the WT quantity (mean ± SEM.; n=3 WT and 3 MAP6 KO mice, respectively). No significant difference was found.

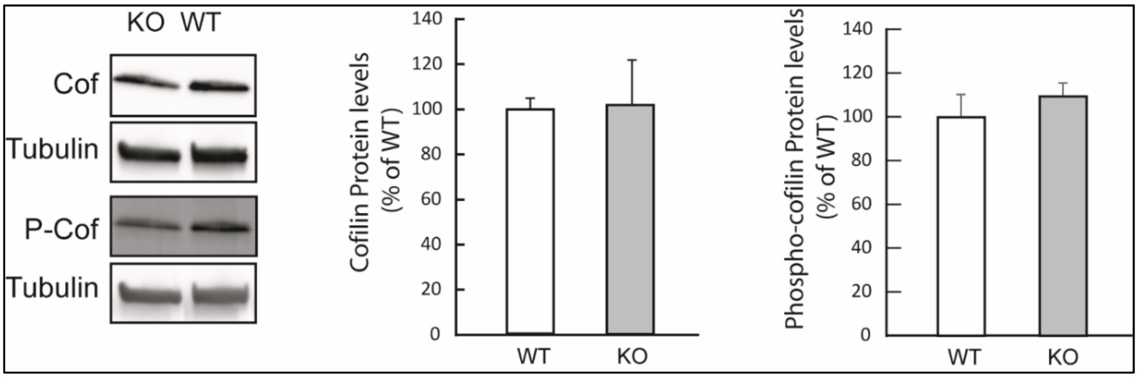

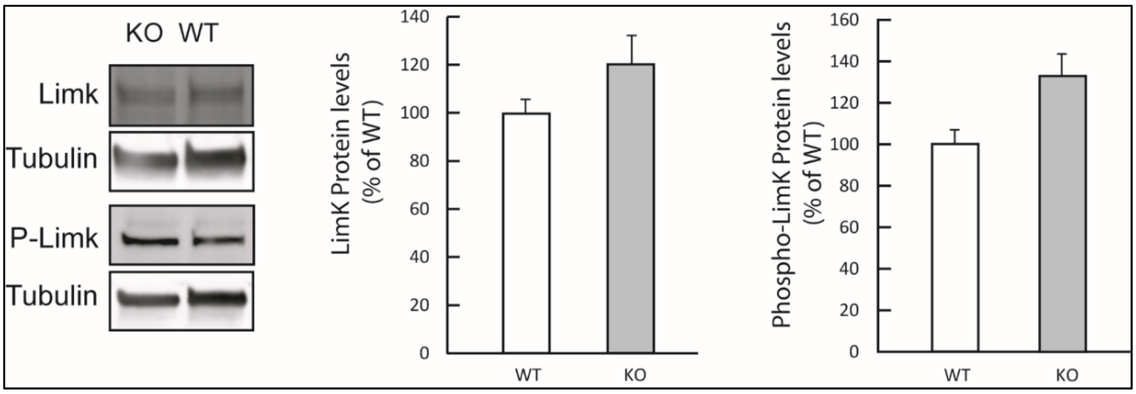

Supplementary Figure S2

Analysis of actin stability at 4°C in MAP6 KO neurons

MAP6 KO neurons at 10 DIV were exposed or not to cold (45 min, 4 °C), permeabilized (30 mM Pipes, 1 mM EGTA, 1 mM MgCl2, 10% glycerol, 1% Triton X100, pH 6.75) for 1 min and fixed in cold methanol (6 min, -20 °C). For immunofluorescence, anti-βactin mAb was used as primary antibody (ab6276 Abcam, 1/200, 1 hour) and revealed with anti-mouse coupled to AF 488 (1/1000, Jackson Immuno-Research Laboratories).

No significant difference was observed.

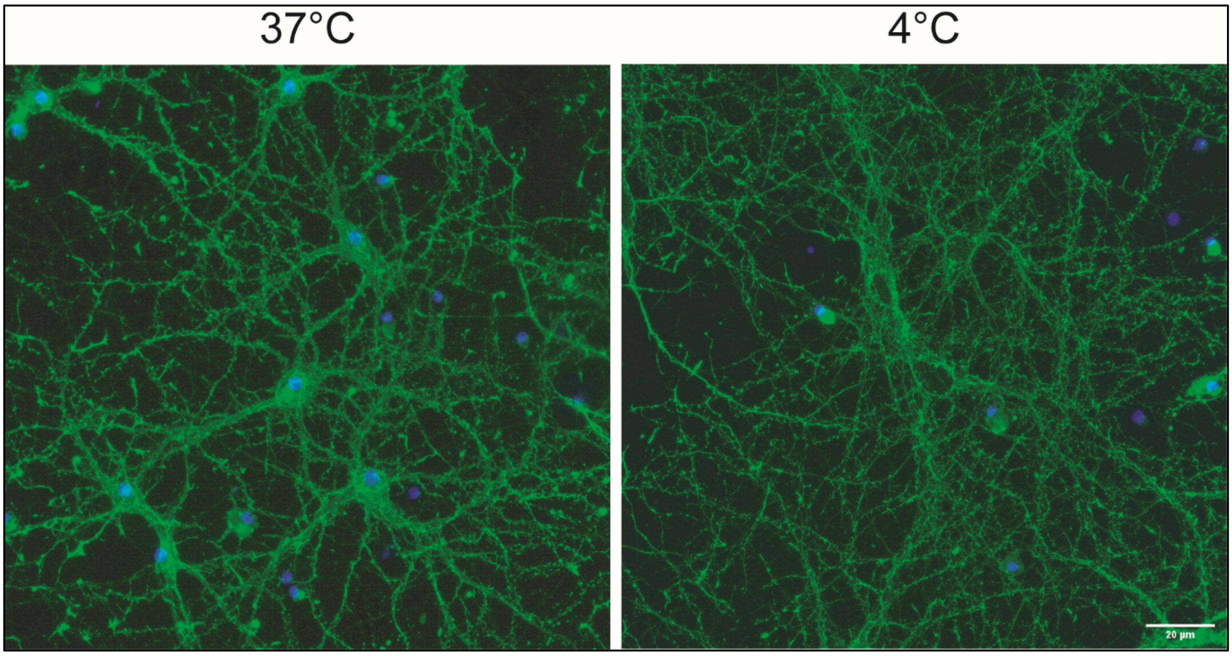

Supplementary Figure S3

Quantification of Pyr1 content in mice brains using LC-MS/MS

LC-MS/MS profiles obtained for untreated and Pyr1 treated mice.

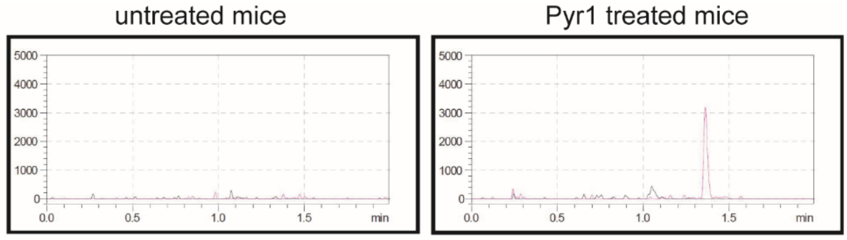

Table 1

Mice number	Pic surface area	Pyr 1 quantity (nmol/brain)	Pyr1 concentration (nM)
1	5653	0,052	119
3	3276	0,03	69
4	4860	0,044	101
7	2142	0,02	46
9	6562	0,06	138
		Average	95 nM

Mice were euthanized by cervical dislocation, brains rapidly dissected, frozen in liquid nitrogen and stored at -80°C until processing. Briefly, each half brain was milled in 400 µL of saline buffer, and Acetonitrile (800 µL) was added to extract Pyr1 compound. The samples were shaken with a vortex for 3 minutes and then placed for 3 minutes in a sonicator. Precipitated proteins and solid residues were sedimented by centrifugation at 15,000g for 5 min at 4°C. The supernatants were transferred in a microplate for analysis by LC-MS/MS (Shimadzu LC-MS 8030) by TechMedIll company (Illkirch, France). The Pyr 1 concentration was calculated using an average brain volume of 430 mm3 as previously determined (Gimenez et al., 2017).

An average concentration of 95 nM of Pyr 1 was found in the mice brains, after 6 weeks of treatment (100 mg/kg/week), whereas no detectable Pyr1 molecule was found in untreated mice.

Gimenez, U., et al., 2017. 3D imaging of the brain morphology and connectivity defects in a model of psychiatric disorders: MAP6-KO mice. Sci Rep. 7, 10308.

Supplementary Figure S4

***Long-Term Potentiation (LTP) assayed in wild type and MAP6 KO mice**

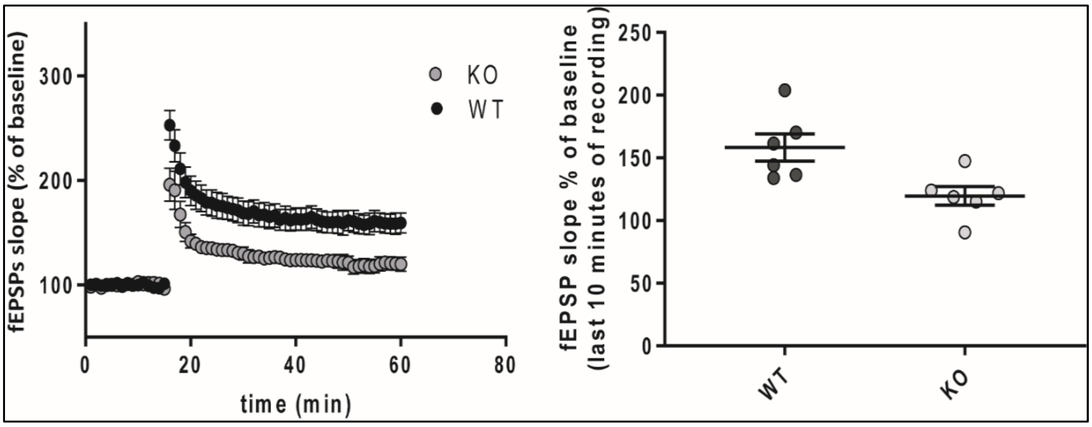

Wild type and MAP6 KO mice LTP were evaluated (as in Fig 2F-G).

Left panel: fEPSP slopes expressed as a percentage of baseline were plotted against time for WT and MAP6 Ko mice brain slices.

Right panel: fEPSP slopes measured over the last 10 min of recordings, expressed as a percentage of the baseline of the fEPSP slope (158.23±10.8 and 119.57±7.4 for wild type and MAP6 KO mice, respectively). Values correspond to mean ± SEM. n=8 slices from 3 wild type mice and n=7 slices from 4 MAP6 KO mice. Mann-Whitney test, p value is 0.0152.

MAP6 KO mice exhibit an altered LTP

***Social interaction of WT and MAP6 KO mice**

Social interaction was assayed using the resident intruder test with a protocol similar to the one used in Fig 3A. (mean ± SEM.; n=7 and n=9 for WT and MAP6 KO mice, respectively).

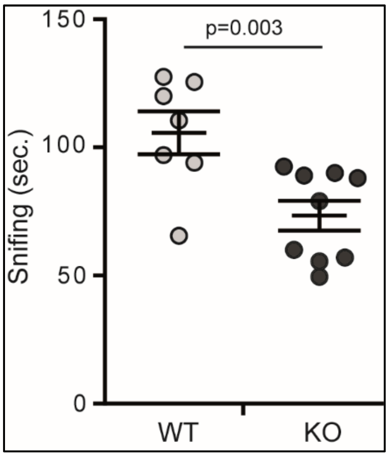

MAP6 KO mice exhibit social withdrawal.

Supplementary Figure S5

Pyr1 treatment does not modify hippocampal neurogenesis

To study neuronal progenitor proliferation, mice were injected with EdU (5-ethynyl-2'-deoxyuridine) (50 mg/kg), 3, 6 and 24h after the last Pyr1 injection. Mice were sacrificed by transcardial perfusion with 4% paraformaldehyde, brains post-fixed for 15 h and cryoprotected in sucrose, before being slicedinto coronal free-floating sections (40 µm). Sections were process as prevouisly described (Jonckheere et al., 2018).

Sections were observed with epifluorescence (Zeiss axioskop). EdU-labeled nuclei were counted within the dentate gyrus every 4 sections throughout the rostro-caudal extent of the hippocampus from bregma -1.34 to -3.38.

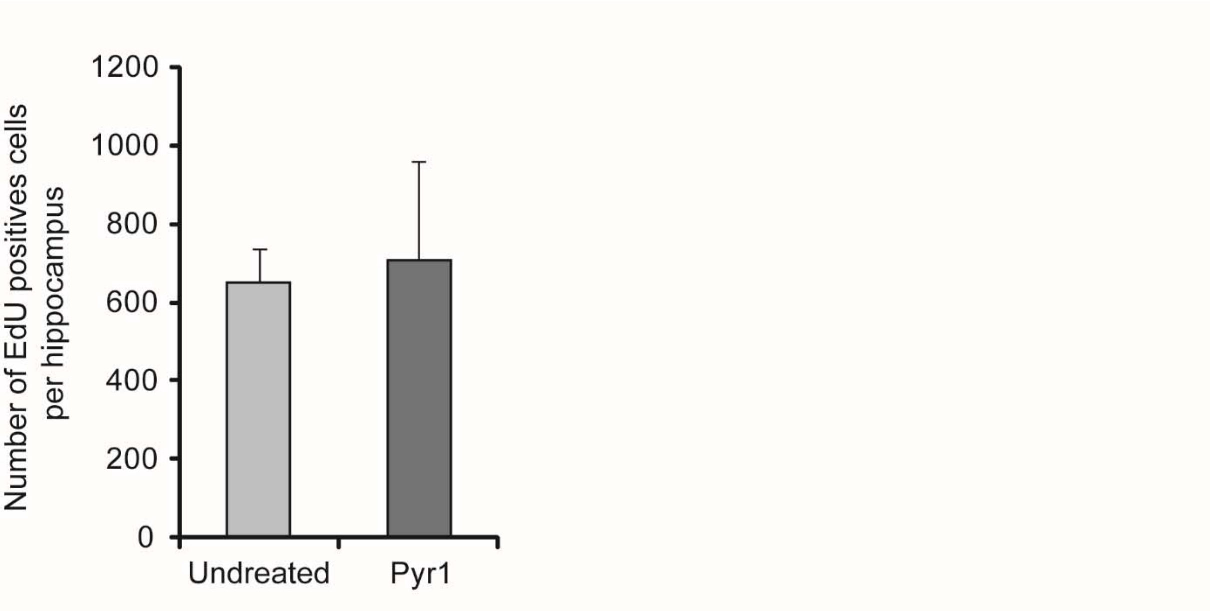

Number of EdU-positive cells per hippocampus (mean ± SEM.; n=7 and n=5 for DMSO- and Pyr1-treated mice, respectively)

These result show that Pyr1 treatment did not modified the number of EdU-positive cells in the sub-granular zone of the hippocampus after chronic treatment (during 6 weeks).

Jonckheere, J., et al., 2018. Short- and long-term efficacy of electroconvulsive stimulation in animal models of depression: The essential role of neuronal survival. Brain Stimul. 11**,** 1336-1347.
